# Social exclusion and its impact on health over the life course: A realist review protocol

**DOI:** 10.12688/hrbopenres.13746.2

**Published:** 2023-12-12

**Authors:** Rikke Siersbaek, Chris O'Donnell, Sarah Parker, John Ford, Sara Burke, Clíona Ní Cheallaigh

**Affiliations:** 1 Clinical Medicine, School of Medicine, Trinity College Dublin, Trinity Centre for Health Sciences, St James's Hospital, Dublin 8, D08 W9RT, Ireland; 2 Safetynet Primary Care, Catherine Mc Auley Education & Research Centre, Dublin 7, D07 A8NN, Ireland; 3 Centre for Health Policy and Management, School of Medicine, Trinity College Dublin, 3-4 Foster Place, Dublin 2, Ireland; 4 Wolfson Institute of Population Health, Queen Mary University of London, London, England, EC1M 6BQ, UK

**Keywords:** Health outcomes, Social exclusion, Marginalisation, Deprivation, Poverty, Life-course, Realist review

## Abstract

**Background:**

Social exclusion is a process whereby certain individuals are born into or pushed to the margins of society and prevented from participating in social, cultural, economic, and political life. People who experience social exclusion are not afforded the same rights and privileges as other population groups. Socially excluded people often experience poorer outcomes in a variety of domains including health, education, employment, and housing than people with socio-economic privilege. People experiencing social exclusion frequently have higher and more complex health needs and poorer access to healthcare than the general population. The aim of this study is to better understand and explain how social exclusion occurs and how it impacts health over the life course.

**Methods:**

A realist review will be undertaken. Data will be collected via a systematic search of databases of peer-reviewed literature and further iterative searches of peer-reviewed and other literatures as needed. The following data bases will be searched: MEDLINE, Embase, CINAHL, and ASSIA, using both indexed subject headings in each database and relevant key words. Grey literature will be searched via Google Scholar and relevant websites of organisations that work with populations affected by social exclusion.

**Conclusion:**

A realist review will be conducted to explain the underlying societal mechanisms which produce social exclusion and related health outcomes in particular contexts affecting excluded population groups across the life course. The study has the potential to inform policy makers and service managers of how and why social exclusion occurs and potential key intervention points to prevent exclusion from happening.

## Introduction

Social exclusion is defined by the United Nations (UN) as ‘a state in which individuals are unable to participate fully in economic, social, political and cultural life, as well as the process leading to and sustaining such a state’ ^1^. Further, according to the UN, social exclusion is ‘multidimensional’ and ‘not limited to material deprivation’ even though ’poverty is an important dimension of exclusion, albeit only one dimension’ ^1^.

In fact, populations experiencing social exclusion often lack adequate access to healthcare, education, social capital, communal connection, housing, services, and more, in addition to often experiencing poverty ^2, 3^. Characteristics associated with social exclusion include substance dependence, homelessness, severe and enduring mental illness, incarceration, institutionalisation, and belonging to certain minority groups (e.g. Travellers, Aboriginal people) ^1, 4^. Often these identities intersect, where people having one experience frequently have other additional experiences of social exclusion ^4^. 

It is well-recognised that health is affected by poverty and other forms of deprivation. As the Marmot Review ^5^ has shown, there is a social gradient in health across society. Those with a high social position have better health outcomes than those with a lower one. Marmot links the cause directly to inequality: ‘Inequalities in health arise because of inequalities in society – in the conditions in which people are born, grow, life, work, and age’ ^5^

However, Marmot also points out that social exclusion is an extreme form of low socio economic status, as he says: ‘social exclusion is deprivation upon stilts’ ^6^. A meta-analysis published in the Lancet in 2018 by Aldridge *et al.*, bore this out when they found a ten-fold increase in all-cause standardised mortality ratios in people who experience social exclusion ^7^.

Similarly, Irish research has demonstrated increased morbidity and mortality among people experiencing social exclusion. Kiernan *et al.* found dramatically increased levels of frailty among people who experience social exclusion in Dublin ^8^. Meanwhile, Ivers and Barry found that the median age at death for men who accessed homelessness services in Dublin was 42 while for women it was even lower at 37 ^9^.

Disadvantage can start before birth with the foundations for physical, intellectual, and emotional child development laid at the earliest stage of life. Additionally, the unequal distribution of resources such as living conditions, education, wealth, networks, supportive family, parenting skills, and social capital across families in society accumulates across the life-course ^5^. Conversely, identifying and acting on systematic differences in health can reduce health inequalities early and can have a profound effect on health outcomes later in life ^5^.

Social exclusion is not a static experience. As mentioned above, it is an intersectional phenomenon where experiences and identities intersect and can amplify each other. Additionally, the expressions and effects of social exclusion accumulate across the life course – a single individual may experience imprisonment, homelessness, and drug dependence across their life course with each potentially compounding the effect of each other deepening social exclusion and causing worse health and life outcomes ^5, 7^. Furthermore, social exclusion is associated with exposures and behaviours which can be damaging to health including excessive alcohol use, smoking of substances including heroin and crack cocaine, injecting drug use, rough sleeping, survival sex, and sex work ^4, 6, 10– 13^.

A body of research has shown which population groups are most likely to experience social exclusion and how it contributes to causing poor health outcomes ^4– 10, 14– 27^. In response, the Inclusion Health approach to research, practice, and policy has emerged with the aim to understand the causes and consequences of social exclusion as well as to characterise and address health inequity arising from it ^4^. However, there is less research explaining the underlying social mechanisms that cause social exclusion to take place at a societal level. The pathways into various and often intersecting expressions of social exclusion such as poverty, homelessness, substance dependency, sex work, etc, have been studied ^28– 33^ but the process by which people who end up in difficult circumstances are then cast as ‘excluded’ within society has not been studied adequately. This study will build on and add to what is already known about stigma, internalised stigma, cultural and social barriers to inclusion, and more, by seeking to explain the underlying causal societal mechanisms that produce social exclusion and its many resulting health effects.

## Protocol

This study will undertake a realist review to understand and explain how social exclusion occurs over the life course. Specifically we will address questions below:

1. How, why, in what circumstances, and for whom is social exclusion produced by underlying, often intersecting, societal mechanisms triggered in particular contexts over the life course resulting in inequitable health outcomes?

Additionally, we will provide recommendations for health and social care policy makers about the contexts in which causal processes occur which create social exclusion.

## Methods

We have chosen to conduct this study as a realist review in the school of Pawson and Tilley ^34– 37^ because it is a useful approach to understanding societal mechanisms which cannot be observed, such as stigma or social exclusion, but which nevertheless produce real life outcomes in specific contexts. To understand the causal mechanisms at play, realist approaches make use of theory and theorising to explain and synthesise data.

**Figure 1.**
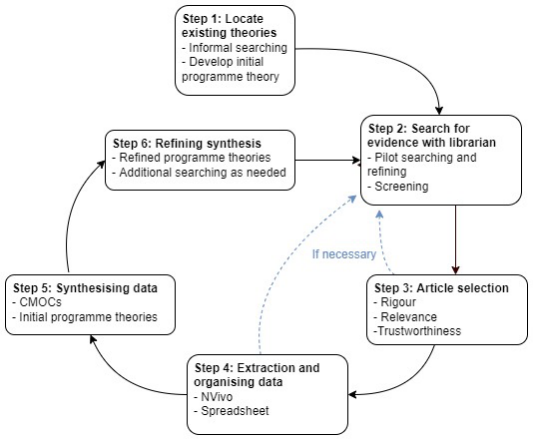
Stages of a realist review.

This realist review will be conducted using the six iterative steps they outline ( Figure 1).

### Initial programme theory

**Figure 2.**
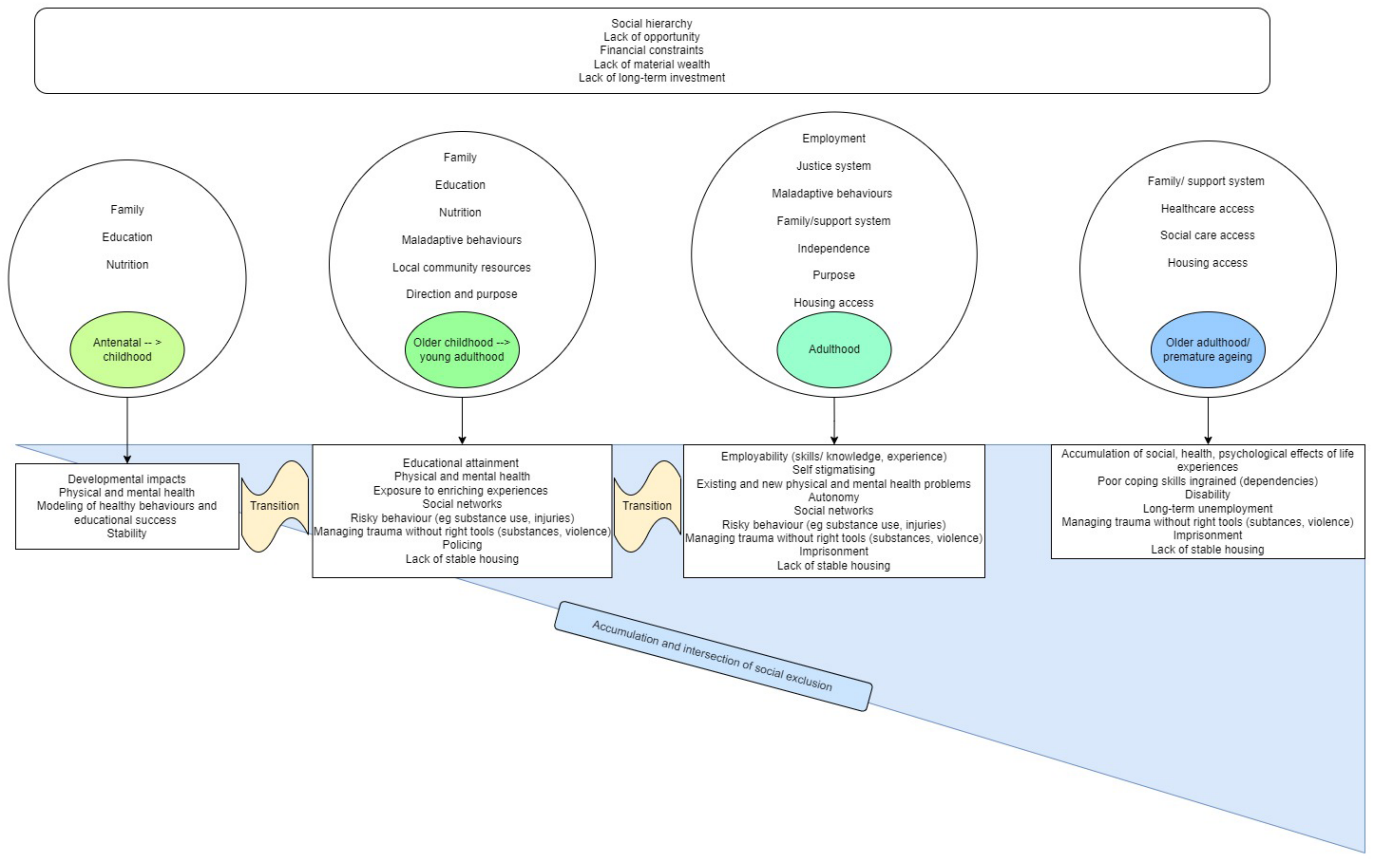
Initial programme theory.

An initial programme theory ( Figure 2) was built based on prior knowledge and robust discussion among the full research team and with input from an expert panel which includes patient and public involvement (PPI). It serves as the starting point for the study showing important factors that create social exclusion over the life course and the cumulative relationship between these. The expert panel includes people with lived experience of social exclusion, healthcare practitioners, and academics who all work in the area of inclusion health and bring various perspectives and expertise on the area, therefore being able to provide robust feedback and support during two formal meetings of the full group and via smaller consultations throughout the research process.

### Searches

A search strategy has been developed by the research team informed by our initial programme theory ( Figure 2), in consultation with the study’s expert panel and a subject librarian. Two search clusters have been identified, one related to ‘social exclusion’ and the second related to the ‘life-course’. We have developed search strings which have been piloted with changes made as needed based on the accuracy and appropriateness of various keywords. The search terms are as follows:

("Social exclu*" OR "social inclu*" OR "social marginal*" OR "social rejection" OR "Low socioeconomic status" OR "low SES" OR Depriv* OR "Severe multiple deprivation" OR "Low income" OR "early life advers*" OR "adverse childhood experiences" OR "ACEs" OR "early life stress" OR "Social class" OR "social factors" OR "Inequalities") AND ("life course" OR "life span" OR "life time") 

A systematic search of peer-reviewed databases will be conducted first of the following databases: MEDLINE, Embase, CINAHL, and ASSIA and using both indexed subject headings in each database and relevant key words. Additional iterative searches of peer-reviewed and other literatures will be conducted when and if needed. Such iterative searches will be performed if a gap in the literature resulting from the first search is apparent and further policy literature, grey literature and/or peer-reviewed literature sources are needed to explore aspects of the research questions. Grey literature will be searched via Google Scholar and relevant websites of organisations that work with populations affected by social exclusion.

### Inclusion and exclusion criteria

Our search will seek to identify information, data, or studies which explain aspects of how social exclusion comes to be and its impact on health outcomes. In particular, for this type of study, we will be identifying sources or parts of sources which show how it happens, to whom, why, and to what extent.

Studies of any design written in English will be included.

Inclusion criteria:

1. Research studies and clinical guidelines of any study design e.g. qualitative, quantitative, mixed methods
2. Review studies
3. Policy documents
4. Non-Governmental Organisation and professional organisation websites
5. Information published in English
6. Any year of publication

Exclusion criteria:

1. Information not published in English

### Data extraction

Articles identified through our systematic searches, and later through iterative searches if needed, will be extracted into Covidence where automatic deduplication will take place. Of these, RS will screen 100 percent of the sources and a random 10 precent sample of the included documents will be independently screened by CO’D and SP for quality control. Potential disagreements will be solved by discussion between RS, CO’D and SP, and the full team if necessary. RS will also review 100 percent of the sources selected for full text review with CO’D and SP again checking 10 percent for consistency.

Papers selected for inclusion will be uploaded to NVivo where RS will code data in accordance with the realist approach as described by Papoutsi *et al.* ^38^ and Tierney *et al.* ^39^. First, high-level conceptual labels will be assigned to identify what a piece of data is telling us about the research question. Then, through iterative rounds of coding, data will be assigned context, mechanism and outcome labels where possible keeping in mind that the same piece of data can act as different parts of different context-mechanism-outcome-configurations (CMOCs). Coding will at first be inductive and deductive with new codes created as needed and subsequent related pieces of data then assigned to codes that have already been created inductively, where appropriate. The second round of coding will be retroductive where a theory-driven realist approach will be used to identify patterns of causality in the data to aid in the creation of CMOCs ^40^.

Data will then be extracted into a word document as context-mechanism-outcome configurations. CMOCs will be written with all supporting data listed after each configuration. CMOCs will then be iteratively refined, combined or rejected while continuously returning to the data to ensure that the synthesis is directly building on the data. Iterative searching will take place if and when needed to fill gaps and refine CMOCs as appropriate. Ultimately the analysis will result in building an overarching programme theory based on the data collected but presented at a higher level of abstraction which is transferrable within similar contexts.

### Assessing quality of evidence

Following the realist approach, we will assess the quality of the data according to the relevance, rigour and trustworthiness of a given piece of data from an included source ^41, 42^. Quality assessment Additionally, risk of bias will be minimised by checking a random 10 percent sample of studies by a second reviewer as discussed above. And finally, quality will be ensured by adherence to the Realist And MEta-narrative Evidence Syntheses: Evolving Standards (RAMESES) publication standards for realist reviews ^41^ which provide guidelines for which relevant and necessary information that should be included in a realist review and allow end users and reviewers to assess the quality and rigour of a review.

### Data synthesis

Typically, realist theory is expressed using the heuristic ‘context + mechanism = outcome construction’ also often written as ‘C+M=O’ or just ‘CMO construction’ or ‘CMOC’. A CMOC is an explanatory device which shows how an outcome is produced when a particular hidden, latent power (mechanism) is triggered in a given context.

In a realist analysis, CMOCs are constructed close to the source material using specific pieces of data directly from the sources included in a review. Crucially data must be configured into explanatory statements describing the causal relationship of a given context, mechanism and outcome to account for the unseen causal action which the theory posits is happening in the data. They are then brought to a higher level of abstraction and are often combined into programme theory/theories which explain patterns of causation removed from the specific data to explain causality in more general terms.

## Study status

The systematic search has been completed and title and abstract screening is currently under way.

## Dissemination

The results of our study will be disseminated primarily through academic networks via a journal article and presentations at academic conferences. We also have plans for a dissemination event for the project which this research is funded under (Health Research Board grant SDAP-2021-029).

## Ethics

Ethical approval is not needed because this study is a synthesis of published literature and no data collection will take place.

## Conclusion

In this research, we will seek to explain underlying societal mechanisms which cause some population groups and individuals to experience social exclusion and negative health outcomes as a result. We will conduct a robust realist review to uncover such societal mechanisms. This study will provide guidance for policy makers by supplying high-level explanations of how social exclusion occurs so they can identify societal causes and key points where interventions can be deployed to improve health outcomes. Ultimately, our work seeks to increase knowledge to prevent social exclusion from occurring and to mitigate its effects on the health of populations and individuals.

## Data Availability

There are no data associated with this article.
