# Supplementary material for: Social exclusion and its impact on health over the life course: A realist review protocol
Source: HRB Open Res. 2023 Dec 12;6:34. Originally published 2023 Jul 24. [Version 2] doi: 10.12688/hrbopenres.13746.2 (PMC10864819; doi:10.12688/hrbopenres.13746.2)
Supplement: Peer review history

# Reviewer report: Reviewer response for version 2

**DOI:** 10.21956/hrbopenres.15153.r37939

**Published:** 2024-05-22

**Authors:** Ana Maria Arias-Uriona

**Affiliations:** 1 Universidad Catolica Boliviana San Pablo, La Paz, La Paz Department, Bolivia

1. **Expanding the Theoretical Framework**: As you mention, "Social exclusion is not a static experience. As mentioned above, it is an intersectional phenomenon" there is a need to explicitly integrate intersectionality as a critical theoretical framework within your study's methodology. This approach acknowledges the multifaceted nature of social identities and how various forms of social stratification (e.g., race, class, gender) intersect to influence individuals' experiences of social exclusion and health disparities. Highlighting this within your protocol can deepen the understanding of how intersecting structural mechanisms contribute to health outcomes.
2. **Refinement of Search Strategy**: Adjust your search strategy to include terms and concepts related to intersectionality and structural mechanisms. This can involve including specific keywords such as "intersectionality," "structural racism," "gender discrimination," "economic inequalities," and "social determinants of health" to capture literature that specifically addresses the interplay of these dimensions in the context of social exclusion and health.
3. **Inclusion Criteria Modification**: Consider revising your inclusion criteria to seek out studies that employ an intersectional analysis or focus on the impacts of intersecting structural mechanisms on health. This ensures that the studies in your review align with the expanded theoretical framework and can provide insights into the complex dynamics at play.
4. **Analysis Approach**: Adapt your data analysis approach to explicitly engage with intersectionality as a lens through which to interpret findings. This might involve identifying and examining patterns in how social identities and structural mechanisms interact to exacerbate or mitigate health disparities associated with social exclusion. This could also mean looking for evidence of how policies or interventions address or fail to address these intersecting factors.

Is the study design appropriate for the research question?

Partly

Is the rationale for, and objectives of, the study clearly described?

Yes

Are sufficient details of the methods provided to allow replication by others?

Partly

Are the datasets clearly presented in a useable and accessible format?

Yes

Reviewer Expertise:

Social exclusión, intersectionality, migration

I confirm that I have read this submission and believe that I have an appropriate level of expertise to confirm that it is of an acceptable scientific standard.

## References

[1] : Intersectionality's Definitional Dilemmas. *Annual Review of Sociology* .2015;41(1) : 10.1146/annurev-soc-073014-112142 1-20 10.1146/annurev-soc-073014-112142

[2] : More than a buzzword: how intersectionality can advance social inequalities in health research. *Critical Public Health* .2020;30(4) : 10.1080/09581596.2019.1584271 494-500 10.1080/09581596.2019.1584271

[3] : Beyond intersectional identities.2023; 10.4324/b23279-12 101-116 10.4324/b23279-12

# Reviewer report: Reviewer response for version 2

**DOI:** 10.21956/hrbopenres.15153.r37395

**Published:** 2024-02-13

**Authors:** Susan Devine

**Affiliations:** 1 College of Public Health, Medical and Veterinary Sciences, James Cook University, Townsville City, Queensland, Australia

Thank you to the authors for their response to my suggested changes. I am satisfied that my comments have been addressed and have nothing further to add. I wish the authors well in their submission.

Is the study design appropriate for the research question?

Yes

Is the rationale for, and objectives of, the study clearly described?

Yes

Are sufficient details of the methods provided to allow replication by others?

Yes

Are the datasets clearly presented in a useable and accessible format?

Yes

Reviewer Expertise:

Social determinants of health. Health promotion.

I confirm that I have read this submission and believe that I have an appropriate level of expertise to confirm that it is of an acceptable scientific standard.

# Reviewer report: Reviewer response for version 2

**DOI:** 10.21956/hrbopenres.15153.r37394

**Published:** 2024-02-13

**Authors:** Esteban Sánchez-Moreno

**Affiliations:** 1 Department of Sociology, Universidad Complutense de Madrid, Madrid, Community of Madrid, Spain

Thank you for the opportunity to read the new version of the article. I do not have any additional comments. My warmest congratulations to the authors for this project, which is of great interest in the field of the study of health and its social determinants.

Is the study design appropriate for the research question?

Yes

Is the rationale for, and objectives of, the study clearly described?

Yes

Are sufficient details of the methods provided to allow replication by others?

Yes

Are the datasets clearly presented in a useable and accessible format?

Not applicable

Reviewer Expertise:

Social determinants of health; social aspects of aging; mental health; socioeconomic inequalities and health

I confirm that I have read this submission and believe that I have an appropriate level of expertise to confirm that it is of an acceptable scientific standard.

# Reviewer report: Reviewer response for version 1

**DOI:** 10.21956/hrbopenres.15037.r36246

**Published:** 2023-10-23

**Authors:** Esteban Sánchez-Moreno

**Affiliations:** 1 Department of Sociology, Universidad Complutense de Madrid, Madrid, Community of Madrid, Spain

Thank you for the opportunity to review this interesting article ("Social exclusion and its impact on health over the life course: A realist review protocol"), which describes in detail the design of a theoretically informed systematic review protocol on the relationship between social exclusion and health. The article is remarkable for the detail with which both the rationale for the review and the procedures for its development are described. In addition, it addresses a research issue of special relevance, since the analysis of the mechanisms that operate in the existence of a social gradient in health in the case of social exclusion is understudied in the literature.This is a well written proposal and I only have a few comments and suggestions.

 ​​​​​​The introduction provides a detailed and rigorous analysis of the concept of social exclusion used, as well as some of the gaps in research on the relationship between social exclusion and health. It should be noted that the authors use a concept of social exclusion focused exclusively on situations of *extreme* social exclusion. This is a legitimate decision which, nevertheless, I believe should be clearly stated. Indeed, recent literature on social exclusion shows that this concept refers to a general process of organization of social inequalities. Therefore, its use is not limited to situations of particular hardship or socioeconomic deterioration, but constitutes a transversal axis around which social inequalities are organized. As a result, the population may be affected by more or less elements of this system of inequality, so that when we use the concept of social exclusion we refer to situations ranging from precarious integration to severe exclusion, passing through different degrees of moderate or mild exclusion. Specifically, this protocol focuses on situations of severe social exclusion, as can be seen from the introduction. 

 The protocol itself is clearly described and well documented. I am concerned that social class is included among the search terms. In the literature on social inequalities in general, and in much of the literature on socioeconomic gradient in health in particular, the concept of social class is clearly differentiated from the concept of social exclusion. Conceptually and theoretically, they refer to differentiated forms of organization of social inequalities. In fact, the processes that could explain the link between social inequalities and health are potentially very different if class or exclusion is considered as the explanatory dimension of inequalities. While in the first case psychosocial approaches seem to be the most appropriate, in the case of exclusion it is possible that neo-materialist explanations may better describe its relationship with health. I suggest bearing in mid this differentiation when analyzing the results of the review.

 ​​​​​I think inclusion criterion number 4 is questionable. This is a source with very different characteristics from the other sources. Is any website eligible? What criteria must the website meet to be considered an eligible source? Will they be quality criteria based on some formal element in the production of information? Please note that consideration I think that the inclusion of this source should be discussed in detail in the paper.

Is the study design appropriate for the research question?

Yes

Is the rationale for, and objectives of, the study clearly described?

Yes

Are sufficient details of the methods provided to allow replication by others?

Yes

Are the datasets clearly presented in a useable and accessible format?

Not applicable

Reviewer Expertise:

Social determinants of health; social aspects of aging; mental health; socioeconomic inequalities and health

I confirm that I have read this submission and believe that I have an appropriate level of expertise to confirm that it is of an acceptable scientific standard, however I have significant reservations, as outlined above.

# Reviewer report: Reviewer response for version 1

**DOI:** 10.21956/hrbopenres.15037.r36239

**Published:** 2023-10-11

**Authors:** Susan Devine

**Affiliations:** 1 College of Public Health, Medical and Veterinary Sciences, James Cook University, Townsville City, Queensland, Australia

Thank you for the opportunity to review the protocol titled “Social exclusion and its impact on health over the life course: A realist review protocol”. This is a well written proposal and I only have a few comments and suggestions.

**Abstract**

 Under the Methods heading, the second sentence could be refined to say….”Data will be collected via a systematic search of *peer-reviewed literature*…… (rather than peer-reviewed databases). It is the literature that is peer-reviewed not the database.

**Introduction**

 First sentence – after United Nations, add the UN abbreviation given that you go onto abbreviate it later in the paragraph.

 In the second paragraph, please use a Capital A for Aboriginal.

**Methods**

 Under the “Initial programme theory” heading – briefly provide some further detail on the expert panel. While the names of panel members are provided in the acknowledgements section, it would be good to have further insight into the level and type of expertise they bring.

 Under “inclusion criteria” –  write NGO out in full. Are there any year limitations on the search?

 Figure 2 is good but I would add “transition” between the last two boxes as well, as this is a very big transition stage. I also wonder if it is worth adding a couple of other things to the last circle, including: loneliness, economic factors, discrimination and ageism and loss of meaningful engagement and social roles, as there is a lot of evidence that supports the importance of these issues. This is just something for the authors to consider.

 Under “Assessing quality of evidence” – write RAMESES out in full and provide a brief explanation of what it is. What aspects of rigor will be considered? You could provide some further detail on how quality will be assessed and how the process of assessing quality will be documented.

Is the study design appropriate for the research question?

Yes

Is the rationale for, and objectives of, the study clearly described?

Yes

Are sufficient details of the methods provided to allow replication by others?

Yes

Are the datasets clearly presented in a useable and accessible format?

Yes

Reviewer Expertise:

Social determinants of health. Health promotion.

I confirm that I have read this submission and believe that I have an appropriate level of expertise to confirm that it is of an acceptable scientific standard, however I have significant reservations, as outlined above.
